# Supplementary material for: Routes to reduction of phosphate by high-energy events
Source: Commun Earth Environ. 2023 Mar 14;4(1):70. doi: 10.1038/s43247-023-00736-2 (PMC11041679; doi:10.1038/s43247-023-00736-2)

Supporting information

Supplmenetary Table 1. Composition of NPR fulgurite glasses. "--" is below detection limits.

	Lechatelierite	SEM Groundmass	LA-ICPMS Groundmass 1	LA-ICPMS Groundmass 2
**SiO_2**	**99.74**	**61.19**	**58.7**	**57.6**
**TiO_2**	**--**	**--**	**0.9**	**0.9**
**Al_2O_3**	**0.10**	**18.67**	**16.2**	**9.9**
**FeO (tot)**	**--**	**--**	**4.1**	**3.5**
**MgO**	**--**	**0.17**	**1.8**	**1.5**
**CaO**	**--**	**19.60**	**16.8**	**24.0**
**Na_2O**	**--**	**--**	**0.8**	**0.7**
**K_2O**	**--**	**--**	**0.2**	**0.1**
**P_2O_5**	**--**	**--**	**0.6**	**1.8**
**Total**	**99.84**	**99.63**	**100**	**100**

Supplementary Figure 1. Iron plaque develops around tree roots in poorly drained soils, such as the soil where the New Port Richey fulgurite formed. These tree roots, when exposed, eventually rot leaving hollow tubes where iron oxide cements quartz sand, as occurs in this tube from Tampa, Florida. The diameter of this tube is 1 cm, and length is 4 cm.

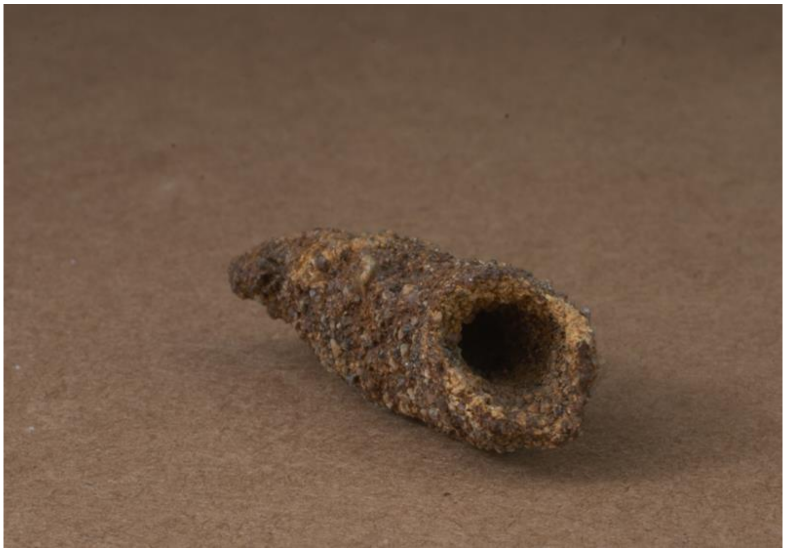

Supplementary Figure 2. BSE image of the region extracted (red circle) for analysis by single-crystal XRD, with the lower panel a blow up of the top panel.

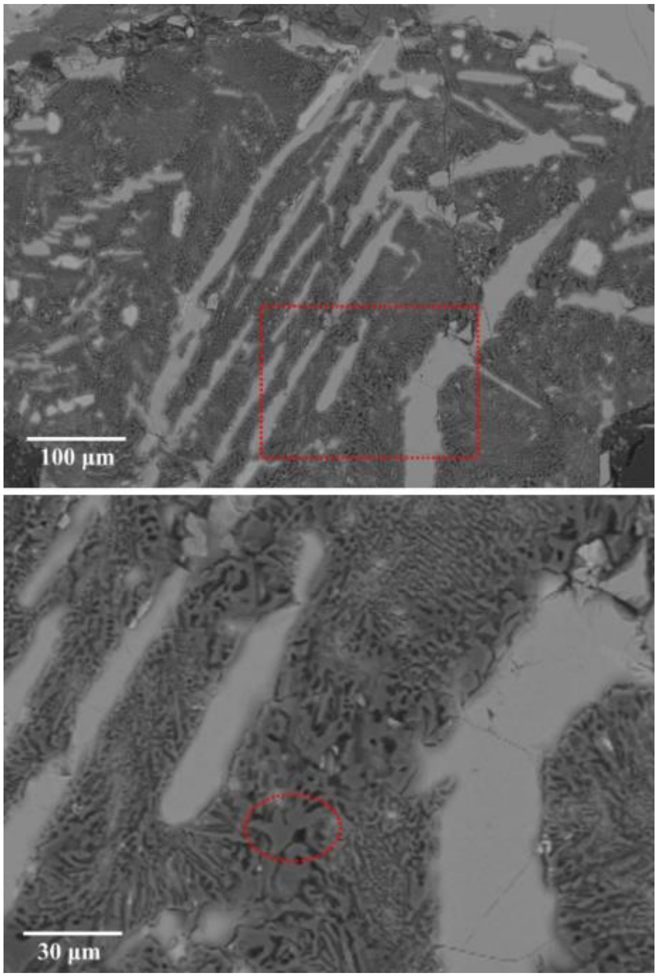

Supplementary Figure 3. Sufficiency of $C_6H_{12}O_6$ (glucose) as a reducing agent to transform (top to bottom) Fe_2O_3 into Fe, Fe_2O_3 and SiO_2 into FeSi, and SiO_2 into Si. In each modeled reaction the equilibrium constant is calculated with respect to one $C_6H_{12}O_6$ molecule.

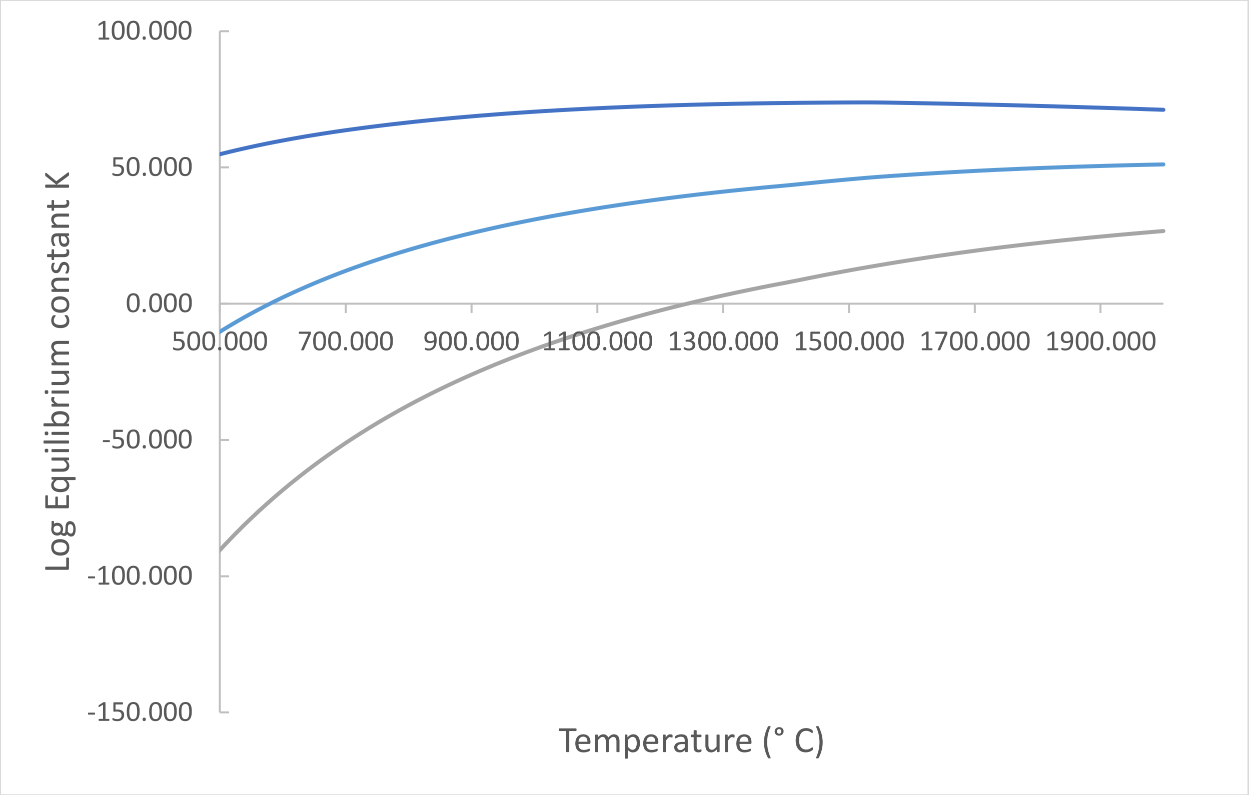

Supplement: Supplementary file 1 — Supplementary Material. [file 43247_2023_736_MOESM1_ESM.pdf]
