# Supplementary material for: Routes to reduction of phosphate by high-energy events
Source: Commun Earth Environ. 2023 Mar 14;4(1):70. doi: 10.1038/s43247-023-00736-2 (PMC11041679; doi:10.1038/s43247-023-00736-2)
Supplement: Supplementary file 2 — Supplementary Video 1 [file 43247_2023_736_MOESM2_ESM.pptx]

## Slide 1
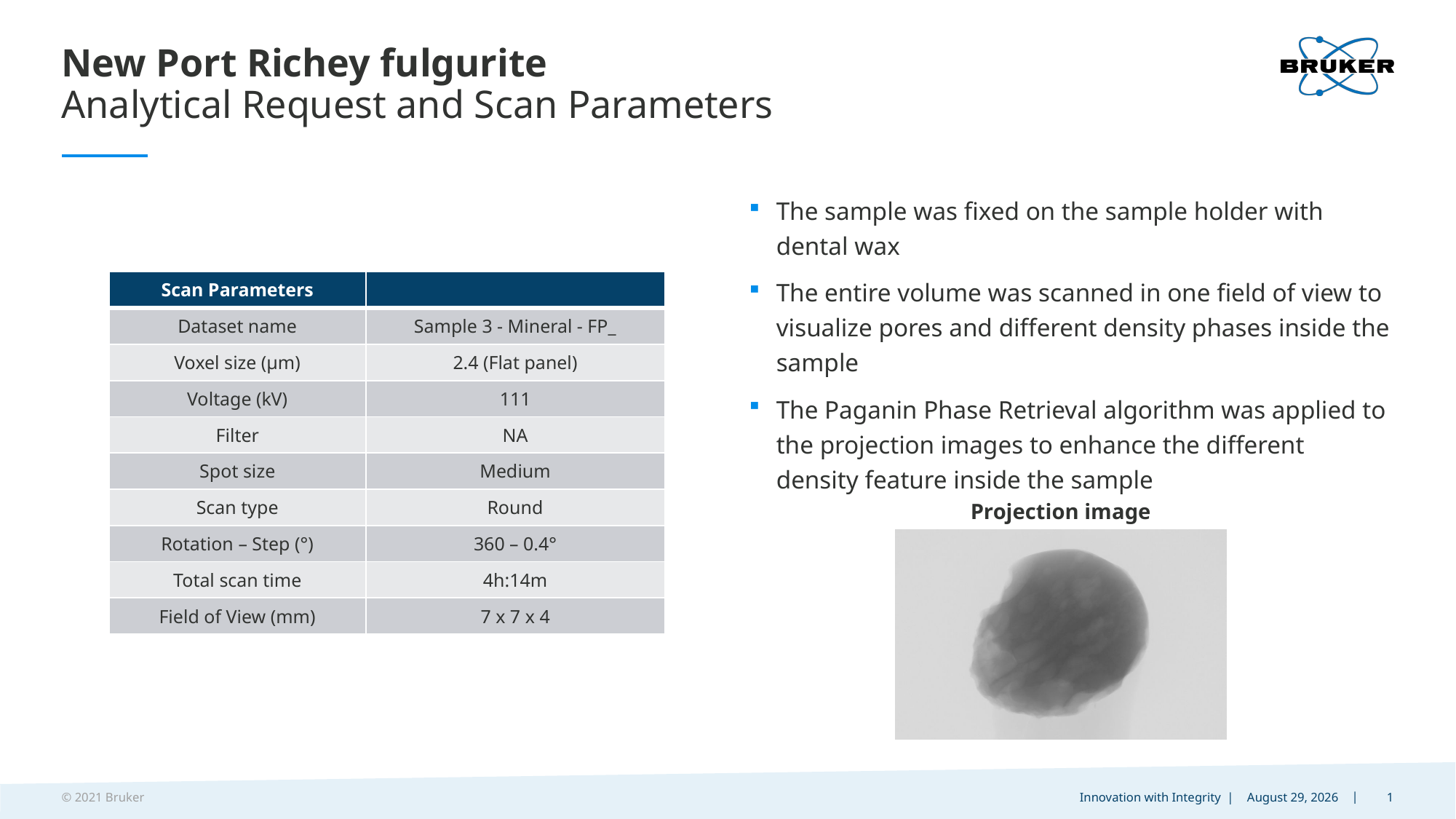

# New Port Richey fulgurite Analytical Request and Scan Parameters
The sample was fixed on the sample holder with dental wax
The entire volume was scanned in one field of view to visualize pores and different density phases inside the sample
The Paganin Phase Retrieval algorithm was applied to the projection images to enhance the different density feature inside the sample
| Scan Parameters | |
| --- | --- |
| Dataset name | Sample 3 - Mineral - FP\_ |
| Voxel size (µm) | 2.4 (Flat panel) |
| Voltage (kV) | 111 |
| Filter | NA |
| Spot size | Medium |
| Scan type | Round |
| Rotation – Step (°) | 360 – 0.4° |
| Total scan time | 4h:14m |
| Field of View (mm) | 7 x 7 x 4 |
Projection image
Innovation with Integrity
26 October 2022
1

## Slide 2
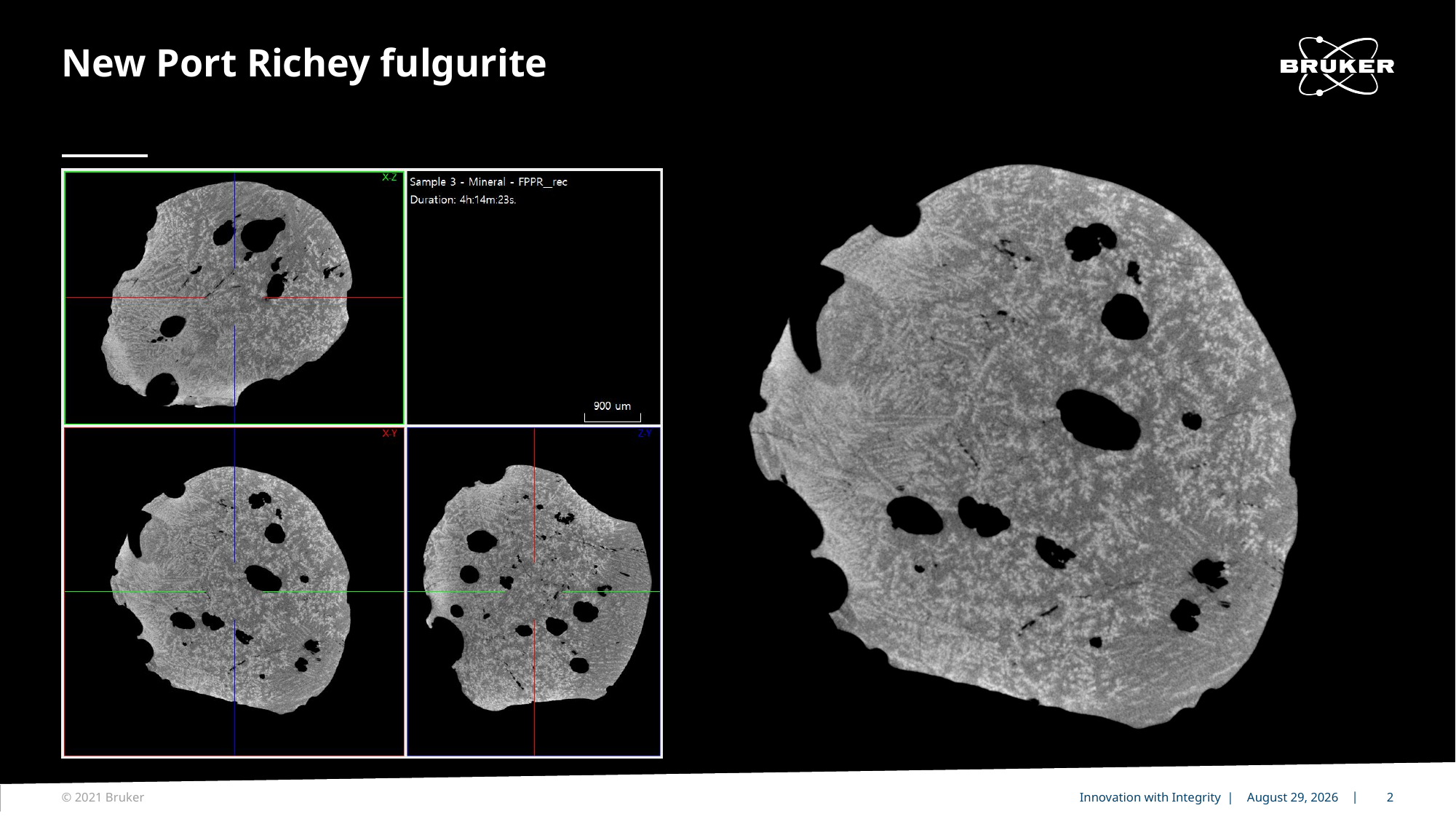

# New Port Richey fulgurite
Innovation with Integrity
26 October 2022
2

## Slide 3
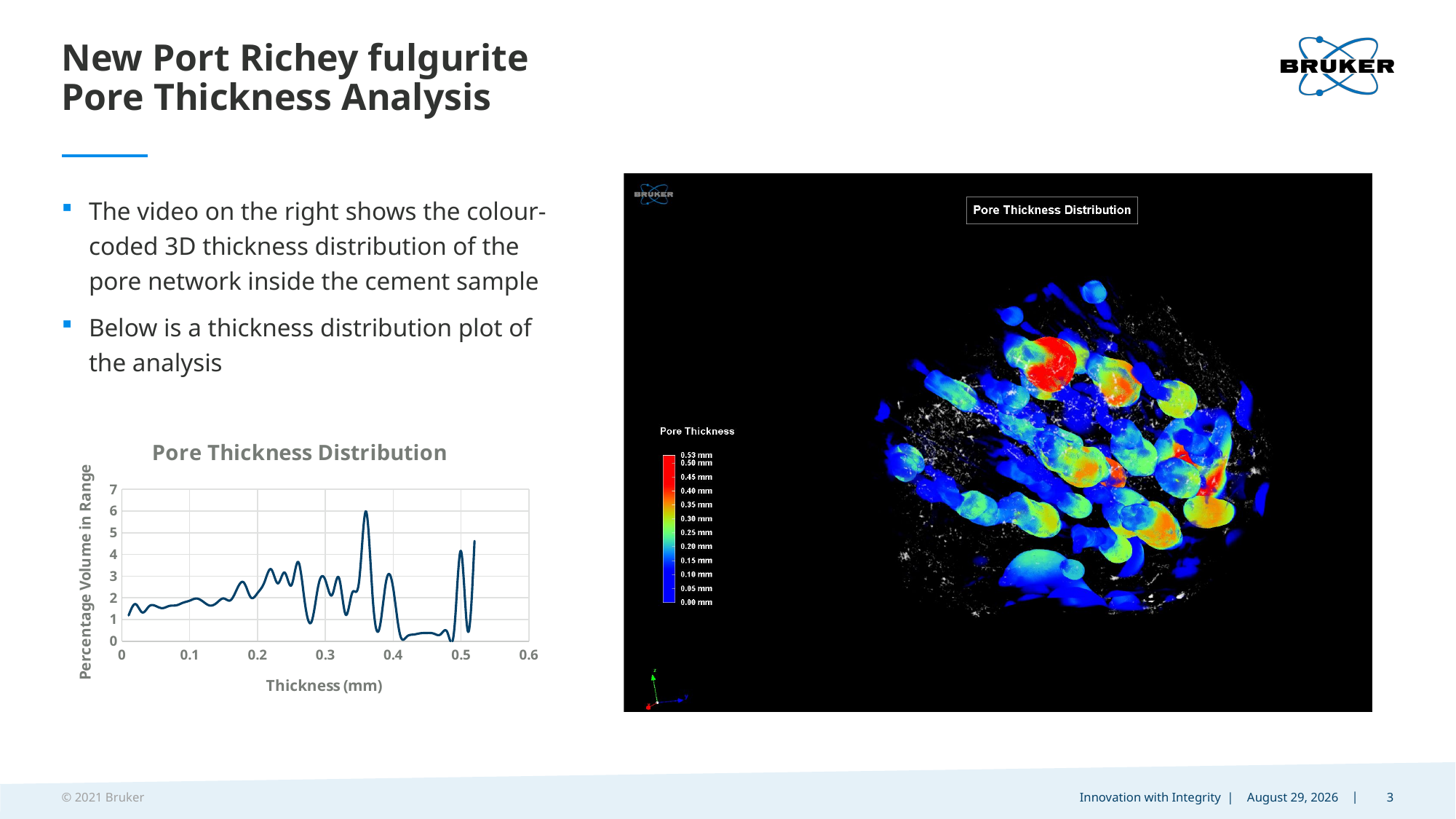

# New Port Richey fulgurite Pore Thickness Analysis
The video on the right shows the colour-coded 3D thickness distribution of the pore network inside the cement sample
Below is a thickness distribution plot of the analysis
### Chart: Pore Thickness Distribution
| Category | |
|---|---|Innovation with Integrity
26 October 2022
3

## Slide 4
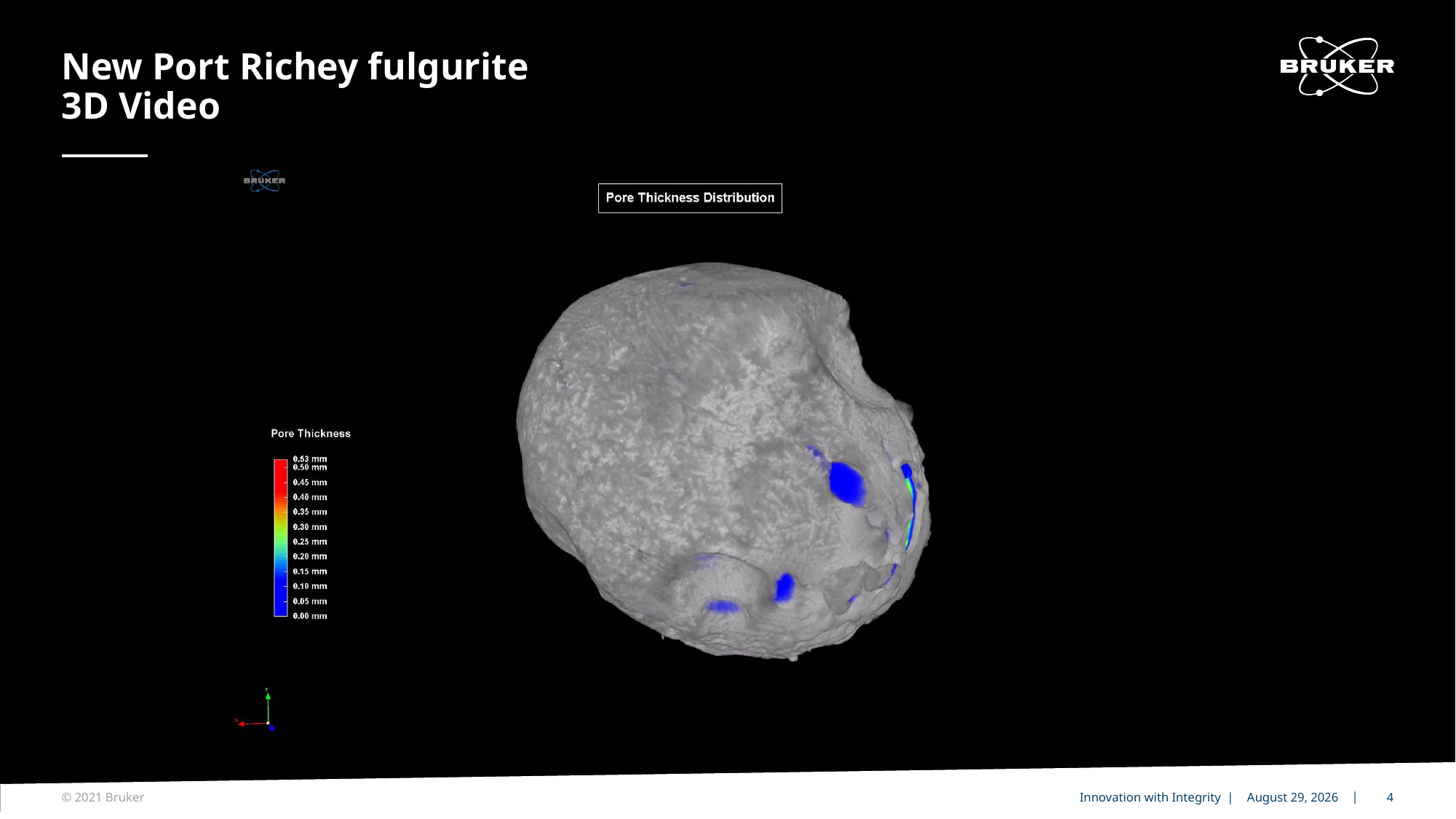

# New Port Richey fulgurite 3D Video
Innovation with Integrity
26 October 2022
4
