# Supplementary material for: Routes to reduction of phosphate by high-energy events
Source: Commun Earth Environ. 2023 Mar 14;4(1):70. doi: 10.1038/s43247-023-00736-2 (PMC11041679; doi:10.1038/s43247-023-00736-2)
Supplement: Supplementary file 4 — Description of Additional Supplementary Files [file 43247_2023_736_MOESM4_ESM.pdf]

Description of Additional Supplementary Files

File Name: Supplementary Video 1

Description: The enclosed power point provides the nanotomography of the New Port Richey metallic spherule. Slide 1 details the scan parameters and methodology. Slide 2 presents sections of the spherule provided through nanotomography. Slide 3 provides the pore thickness of the spherule. Slide 4 provides the animation of the spherule with pore spaces shown through rotation.
